# cAMP-dependent activation of protein kinase A attenuates respiratory syncytial virus-induced human airway epithelial barrier disruption

**DOI:** 10.1371/journal.pone.0181876

**Published:** 2017-07-31

**Authors:** Fariba Rezaee, Terri J. Harford, Debra T. Linfield, Ghaith Altawallbeh, Ronald J. Midura, Andrei I. Ivanov, Giovanni Piedimonte

**Affiliations:** 1 Pediatric Research Center and Pediatric Institute, Cleveland Clinic Children’s, Cleveland, Ohio, United States of America; 2 Pathobiology Department, Lerner Research Institute, Cleveland, Ohio, United States of America; 3 Biomedical Engineering Department, Lerner Research Institute, Cleveland, Ohio, United States of America; 4 Department of Human and Molecular Genetics, Virginia Institute of Molecular Medicine, Virginia Commonwealth University, Richmond, Virginia, United States of America; Emory University School of Medicine, UNITED STATES

## Abstract

Airway epithelium forms a barrier to the outside world and has a crucial role in susceptibility to viral infections. Cyclic adenosine monophosphate (cAMP) is an important second messenger acting via two intracellular signaling molecules: protein kinase A (PKA) and the guanidine nucleotide exchange factor, Epac. We sought to investigate effects of increased cAMP level on the disruption of model airway epithelial barrier caused by RSV infection and the molecular mechanisms underlying cAMP actions. Human bronchial epithelial cells were infected with RSV-A2 and treated with either cAMP releasing agent, forskolin, or cAMP analogs. Structure and functions of the Apical Junctional Complex (AJC) were evaluated by measuring transepithelial electrical resistance and permeability to FITC-dextran, and determining localization of AJC proteins by confocal microscopy. Increased intracellular cAMP level significantly attenuated RSV-induced disassembly of AJC. These barrier-protective effects of cAMP were due to the activation of PKA signaling and did not involve Epac activity. Increased cAMP level reduced RSV-induced reorganization of the actin cytoskeleton, including apical accumulation of an essential actin-binding protein, cortactin, and inhibited expression of the RSV F protein. These barrier-protective and antiviral-function of cAMP signaling were evident even when cAMP level was increased after the onset of RSV infection. Taken together, our study demonstrates that cAMP/PKA signaling attenuated RSV-induced disruption of structure and functions of the model airway epithelial barrier by mechanisms involving the stabilization of epithelial junctions and inhibition of viral biogenesis. Improving our understanding of the mechanisms involved in RSV-induced epithelial dysfunction and viral pathogenesis will help to develop novel anti-viral therapeutic approaches.

## Introduction

The airway epithelial barrier functions as the front line of host defense against airborne threats. The integrity of this barrier is essential for the regulation of innate immunity of the lungs, but such barrier integrity is known to be disrupted by a number of environmental stimuli. Barrier properties of the airway epithelium are primarily determined by the Apical Junctional Complex (AJC) composed of tight junctions (TJ) and adherens junctions (AJ) [1]. It has been shown that the AJC not only regulates the structural integrity of tissues, but also the selective paracellular permeability, cellular differentiation, migration, and signal transduction [2]. Recent genome-wide association studies have uncovered a link between gene polymorphisms in several junctional proteins such as *PCDH1* (Protocadherin 1 gene) and *CDHR3* (encoding cadherin-related family member 3) with asthma [3, 4], as well as increased risk for severe viral infections [5]. While viruses are responsible for the majority of respiratory illnesses in children [6], mounting evidence indicates that increased permeability of the airway epithelial barrier is a common manifestation of lower respiratory tract viral infections that may significantly contribute to the development of mucosal inflammation [7]. These findings highlight the importance of studying the effects of viral infections on structure and functions of the airway epithelial barrier.

Respiratory syncytial virus (RSV) is the most common respiratory virus that causes lower respiratory tract infection and inflammation. Historically considered a pediatric disease, RSV infection is now recognized as an important source of morbidity and mortality in elderly and high-risk adults [8]. Investigations in cell culture and animal models have shown long-term inflammation post-RSV infection [9–13]. In addition, human studies have shown strong associations between RSV, persistent wheezing, and childhood asthma [14–16]. Despite extensive research, current treatment strategies for RSV are largely supportive. Palivizumab, a monoclonal antibody approved for RSV prophylaxis in high-risk infants, has only moderately decreased hospital admissions due to RSV infection [17, 18].

The pathogenic mechanisms of RSV infection remain poorly understood. Specifically, little is known about the effects of RSV on the structure and functions of the airway epithelial barrier. Some previous studies [19, 20] demonstrated that RSV infection increases permeability of cultured bronchial epithelial cell monolayers by triggering protein kinase D-dependent TJ disassembly, but others showed differently [21, 22]. Because such epithelial barrier dysfunction could significantly accelerate RSV-induced airway inflammation, it is important to understand cellular mechanisms that either attenuate disassembly or promote recovery of the airway epithelial AJC during viral infections.

Cyclic adenosine monophosphate (cAMP) is an important second messenger required for many critical homeostatic cellular functions [23]. cAMP is known to activate two major signaling mechanisms, one involving protein kinase A (PKA) and the other involving Epac, a guanine-nucleotide exchange factor for Rap1 small GTPase [24]. The effects of cAMP signaling on intercellular junctions have been extensively studied in vascular endothelium [25–27]. These studies demonstrate that elevated cAMP level in endothelial cells could have either barrier-protective or barrier-disruptive effects depending on external stimuli and intracellular sites of cAMP accumulation [28]. Forskolin is produced by the Indian *Coleus forskoliii* plant and it is known to activate adenyl cyclase, increasing the level of intracellular cAMP. The efficacy of oral forskolin for asthma prophylaxis was trialed during two small, single-center studies, and found to reduce asthma attacks, which was thought to act through cAMP smooth muscle relaxation [29, 30]. However, neither of these studies reported the adverse effects, which include tachycardia, lowering blood pressure, increasing risk of bleeding and interaction with gluconeogenesis [31–33].

There are very few studies on the effect of cAMP signaling in airway epithelial cells, and more importantly, the effect of cAMP signaling on RSV-infected epithelium has not been investigated. The goal of this study was to test the hypothesis that an elevated cAMP level could prevent AJC disruption and increased permeability caused by RSV infection in model airway epithelial cell monolayers. We report a barrier-protective role for cAMP in the infected epithelial cells that involves multiple mechanisms, such as stabilization of AJ and TJ structure, attenuation of RSV-induced rearrangement of the cortical cytoskeleton, and inhibition of viral propagation.

## Materials and methods

### Antibodies

The following primary monoclonal antibodies (mAbs) and polyclonal antibodies (pAbs) were used to detect junctional and signaling proteins by immunofluorescence labeling and immunoblotting: anti-occludin, anti–ZO-1, and anti–E-cadherin mAb (Thermo-Fisher Scientific, Waltham, MA); anti–β-catenin mAb (BD Bioscience, San Jose, CA), Anti-claudin 1 and 4 pAbs (Abcam, Cambridge, UK), anti-cortactin mAb (p80/85 clone 4F11, EMD Millipore, Billerica, MA), anti-cortactin pAb (H222), anti-phospho-CREB (Ser133; 87G3) rabbit mAb, and anti-CREB (48H2) rabbit mAb (Cell Signaling Technologies, Danvers, MA), anti-GAPDH mAb (6C5, Abcam, Cambridge, MA). Fluorescently labeled phalloidin 488 (Thermo-Fisher Scientific) was used to visualize actin filaments. Anti-rabbit and anti-mouse secondary antibodies conjugated to Alexa-488 or Alexa-568 dyes were obtained from Thermo-Fisher Scientific. Mouse and rabbit secondary HRP-conjugated antibodies were purchased from GE Healthcare (Pittsburgh, PA).

### Chemical and reagents

Forskolin and H-89 were obtained from Sigma-Aldrich (St. Louis, MO); 8-Bromo-cAMP and 8CPT-2Me-cAMP were purchased from Tocris Bioscience (Bristol, UK). High-molecular-weight polyI:C (was purchased from InvivoGen (San Diego, CA). Fluorescein-conjugated 3-kDa dextran was obtained from Thermo-Fisher Scientific.

### Airway epithelial cell culture

16HBE14o- human bronchial epithelial cells (a gift from Dr. Dieter C. Gruenert, University of California San Francisco) were cultured in collagen-coated transwells or 24-well plastic plates as previously described [19, 34]. Primary normal human bronchial epithelial (NHBE; Lonza, Basal, Switzerland) cells from normal and diseased donors were expanded for 1–2 passages. Cells were trypsinized and seeded on a collagen coated 0.32 cm^2^ insert and grown in defined media (Gentamicin, Amphotericin B, and Pen/Strep added to Dulbecco’s Minimal Essential Medium (DMEM) with Ham’s F12) and differentiated at the air-liquid interface as evidence by completely dry apical surfaces, and by transepithelial electrical resistance greater than 1500 Ω/cm^2^ surface area, observed ciliary movement, and mucus production [34].

### Viral infection of epithelial cell cultures

Wild-type RSV strain A2 (RSV A2) stocks were grown as previously described [19]. Polarized human airway epithelial cells were infected apically with RSV A2 diluted in DMEM at a multiplicity of infection (MOI) of 0.5. Control cell monolayers received DMEM alone. In some experiments, we used rgRSV244 (RSV derived from RSV A2 expressing the green fluorescent protein gene), a kind gift from Drs. Mark Peeples (Nationwide Children’s Hospital Research Institute, Columbus, OH) and Peter Collins (National Institutes of Health, Bethesda, MD), as described previously [20, 35].

### Transepithelial electrical resistance and dextran permeability assay

Epithelial permeability to small ions was evaluated by transepithelial electrical resistance (TEER) measurements of normal and RSV-infected cell monolayers using an EVOMX voltohmmeter (World Precision Instruments, Sarasota, FL). Only well-differentiated cell monolayers with TEER >500 Ω x cm^2^ were used in these experiments. The presented data are calculated as percent changes compared to either vehicle-treated controls or time zero of viral infection.

Epithelial permeability for larger molecules was evaluated by measuring transmonolayer fluxes of fluorescein isothiocyanate (FITC)-conjugated dextran 3 kDa (Invitrogen), as previously described [34, 36, 37]. Briefly, epithelial cell monolayers growing on transwell filters were apically exposed to 0.5 mg/mL of FITC-labeled dextran in phosphate-buffered saline (PBS). After 30 minutes of incubation, PBS samples were collected from the lower chamber, and FITC fluorescence intensity was measured using a FlexStation 3 plate reader (Molecular Devices, Sunnyvale CA) with excitation and emission wavelengths of 485 and 544 nm, respectively. After the value of dextran-free PBS was subtracted from each measurement, the concentration of FITC-dextran was calculated using Prism 5.03 software (GraphPad, La Jolla, CA) based on a plotted standard curve prepared via serial dilutions of the stock solution of FITC-labeled dextran in PBS.

### Immunofluorescence staining of junctional proteins, and confocal microscopy

Epithelial cell monolayers grown on transwell inserts were subjected to different fixation protocol. To label the AJC proteins and cortactin, cells were fixed with either 100% cold methanol or ethanol, respectively. To visualize actin filaments and CREB proteins, the cells were fixed in 4% neutral-buffered paraformaldehyde (PFA), with subsequent permeabilization with 0.05% Triton-X100. The fixed cells were incubated with specific primary antibodies, followed by incubation with Alexa Fluor-labeled secondary antibodies. Nuclei were stained with DAPI (Sigma-Aldrich).

Immunofluorescently-labeled cell monolayers were examined using an Olympus FluoView 1000 confocal microscope (Olympus America, Center Valley, PA) with a 100X U Plan S Apo 1.4 NA oil objective. The Alexa Fluor 488 and 568 signals were imaged sequentially in frame-interlace mode to eliminate cross talk between channels. The images were processed using Olympus FV10-ASW 2.0 Viewer software and Adobe Photoshop. Images shown are representative of at least 3 independent experiments, with multiple images taken per slide.

### Protein electrophoresis and immunoblotting

After indicated treatments of cell monolayers in cell culture plates, cells were washed with cold PBS and lysed in RIPA buffer (Santa Cruz Biotechnologies, Dallas, TX). Protein concentration was quantified with a bicinchoninic acid (BCA) protein assay (Pierce Thermo Scientific, Waltham, MA). Equal concentrations of protein from each sample were resolved on a SDS-PAGE and transferred to a PVDF membrane (Bio-Rad, Hercules, CA). Membranes were blocked with non-fat dry milk, or in cases of phosphor antibodies with bovine serum albumin (BSA), and probed with primary antibodies overnight followed by secondary antibodies. Membranes were exposed to ECL (GE Healthcare) and protein bands were detected using X-ray film. GAPDH was used as a lane protein loading control.

### Extraction of RNA and quantitative real-time polymerase chain reaction analysis

Total RNA was extracted from pretreated monolayers in cell-culture plates using E.Z.N.A total RNA kit (OMEGA bio-tek, Norcross, GA) with additional DNase digestion (Omega). cDNA was synthesized using qScript cDNA Synthesis Kit (Quanta Bioscience, Gaithersburg, MD) and was amplified by real-time PCR with an iQ5 multicolor Real-Time PCR Detection System (Bio-Rad) using SYBR Green fastmix (Quanta Bioscience) and primers targeting the 87 bp sequence of the RSV strain A2 genome, which encodes for the viral fusion (F) protein (5’ CACCCTGTTGGAAAC 3’ and 5’ CTCTGTCAGTTCTTG 3’-from Sigma-Aldrich). Transcript expression was normalized using glyceraldehyde 3-phosphate dehydrogenase (GAPDH) as the housekeeping gene. The relative change in gene expression was calculated using the formula: % change = 2^^-(ΔΔCt)^ = 2-ΔCt(treated samples)- ΔCt(control samples) where ΔCt = -Ct (detected gene)-Ct(GAPDH) and Ct is the threshold number.

### cAMP measurement

Intracellular cAMP concentration in the clarified lysates was determined using CatchPoint cAMP kit (Molecular Devices; Sunnyvale, CA) as per manufacturer’s instructions.

### Statistical analysis

Data were analyzed using Prism software (GraphPad, San Diego, CA) and Microsoft Excel. Data are representative of three or more experiments and are presented as means ± SEMs. Data were evaluated statistically with ANOVA or the Student’s t-test, with Bonferroni correction for multiple comparisons. Significance was considered at a *P* value of less than 0.05.

## Results

### Forskolin markedly attenuates RSV-induced AJC disassembly

**Fig 1:**
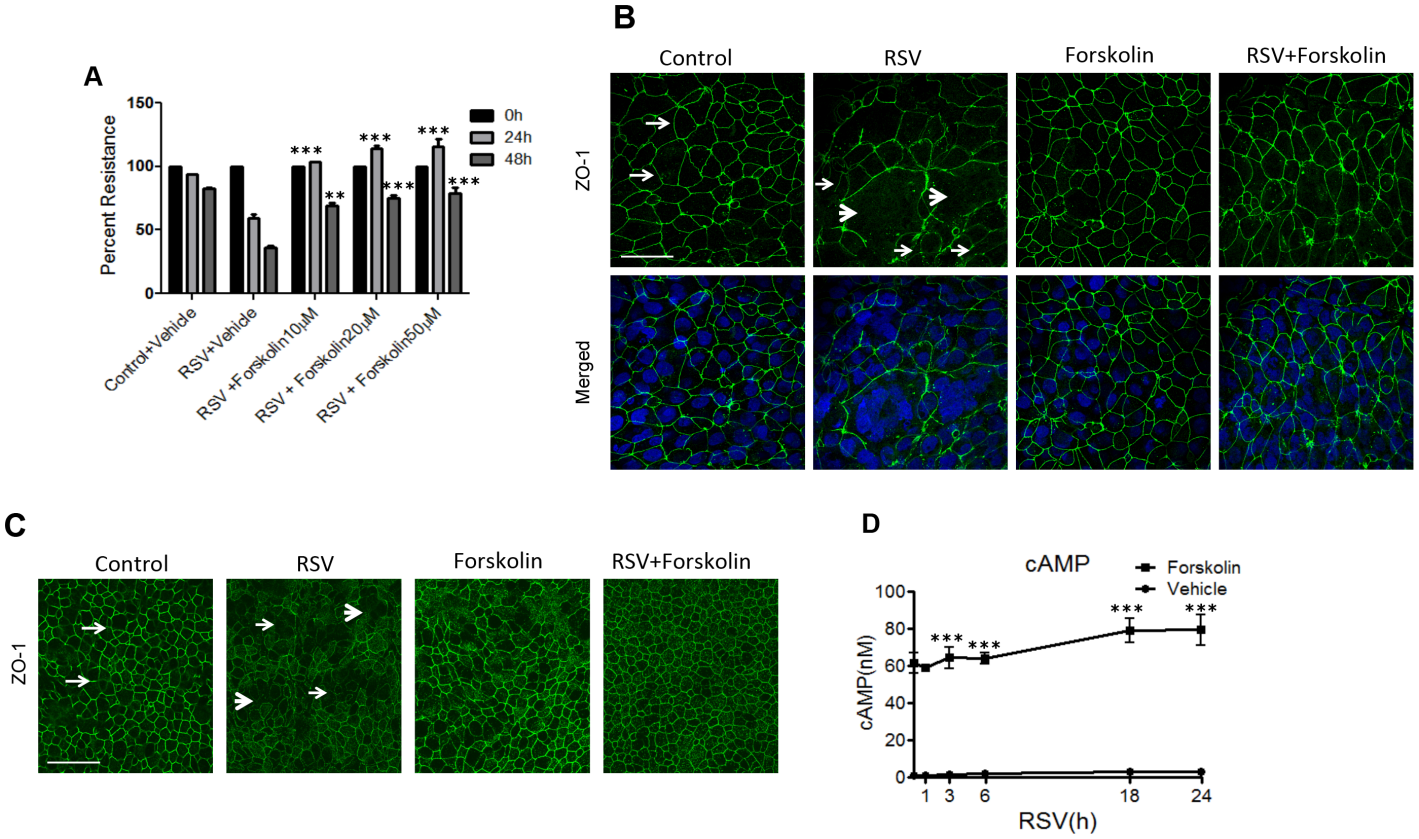
Forskolin attenuates RSV-induced epithelial tight junction disassembly by increasing the intracellular cAMP level. (A) Confluent epithelial cell monolayers were infected with RSV (MOI, 0.5) for 48 h in the presence or absence of forskolin (10–50 μM,). TEER was measured at indicated time points. (B) Confluent epithelial cell monolayers were infected with RSV (MOI, 0.5) for 48 h in the presence or absence of forskolin (20 μM). Tight junction protein, ZO-1 (green), was visualized by immunofluorescence labeling and confocal microscopy. The nuclei were counterstained with DAPI (blue). Note the characteristic “chicken wire” appearance of ZO-1 in control non-infected cells (arrows), and ZO-1 translocation into cytoplasmic dot-like structures in RSV-infected cells (thin arrowheads). Also, note the syncytia formation in RSV-infected cells (thick arrowheads). Scale bar, 40 μm. (C) Primary human bronchial epithelial cells were infected with RSV (MOI, 2) for 48 h in the presence or absence of forskolin followed by visualizing ZO-1 by immunofluorescence labeling and confocal microscopy. Arrows demonstrate normal junction formation, and thin arrowheads indicate the disappearance of TJ ZO-1 staining in the RSV-infected cell monolayer and thick arrowheads shows syncytia formation. Scale bar, 40 μm (D) Cells were infected with RSV (MOI, 0.5) for 24 h, followed by forskolin treatment (20 μM) for 15 min and subsequent measurement of the cAMP concentration in the total cell lysates. Each image and graph is representative of at least 3 independent experiments. Data is presented as mean ± SEM **, *P<* 0.01 and ***, *P<* 0.001 as compared to RSV-infected vehicle-treated cells.

To test the hypothesis that increased intracellular cAMP level could protect the epithelial barrier from disruption caused by RSV infection, polarized airway epithelial cells were infected with RSV strain A2 at a 0.5 MOI in the presence or absence of forskolin, a known activator of adenylyl cyclase, which is responsible for cAMP production. In agreement with our previously published data [19], RSV caused a marked decrease in TEER at 24 and 48 h of viral infection (Fig 1A), thereby indicating disruption of the epithelial barrier. Two different concentrations of forskolin (20 and 50 μM) consistently attenuated the RSV-induced barrier breakdown. Based on these results, the forskolin concentration of 20 μM was chosen for subsequent experiments. In order to examine if such forskolin-dependent preservation of the epithelial barrier is mediated by its effects on the AJC, we next visualized TJ organization in control and RSV-infected epithelial cell monolayers. Immunofluorescence labeling of an essential TJ protein, zonula occludens (ZO)-1, demonstrated a normal ‘chicken wire’ TJ pattern in control cell monolayers (Fig 1B, arrows). This pattern was significantly disrupted after 48 h of RSV infection reflecting TJ fusion during formation of multicellular syncytia (Fig 1B, thick arrowheads), along with intracellular accumulation of ZO-1 (Fig 1B, thin arrowheads). Incubation with forskolin (48 h) did not affect ZO-1 labeling in control epithelial cell monolayers, but prevented RSV-induced alterations in ZO-1 labeling (Fig 1B). Together, these data indicate that forskolin attenuates disruption of TJ structure and increased paracellular permeability in bronchial epithelial cells caused by RSV infection.

16HBE14o- cells have been shown to exhibit well-defined AJCs and appear morphologically similar to airway epithelial cells *in vitro* [19, 34, 38, 39]. To ensure physiological relevance of the results obtained with immortalized 16HBE14o- bronchial epithelial cells, we also used primary normal human bronchial epithelial (NHBE) cells cultured at an air-liquid interface and differentiated into a mucociliary phenotype. NHBE cell monolayers were infected apically with RSV for 48 h in the presence and absence of forskolin. Under the air-liquid conditions, NHBE cells formed tight junctions (Fig 1C arrows). Similar to 16HBE14o- cells, RSV induced a substantial TJ disassembly in NHBE cells, which was manifested by the disruption of ZO-1 labeling (Fig 1C, thin arrowheads), and syncytia formation (thick arrowheads). This junctional disruption was completely prevented by forskolin treatment (Fig 1C). Because 16HBE14o- cells exhibited a similar response to RSV infection as differentiated human primary cells, we utilized those for our subsequent experiments.

Given the fact that forskolin is a potent inductor of cAMP production, we next examined whether the protective effects of forskolin on airway epithelium is associated with an increase in intracellular cAMP. Polarized bronchial epithelial cells were infected with RSV (MOI, 0.5) for varying lengths of time (1–24 h), followed by stimulation with forskolin (20 μM) for 15 minutes. At all tested times, intracellular cAMP was increased by 30–40 fold from baseline after 15 minutes added forskolin to the RSV-infected cell cultures (Fig 1D). Note that mock-infected cultures had similar cAMP levels as RSV infected cells, and RSV infection alone did not alter the baseline intracellular cAMP levels (Fig 1D).

### Treatment with a stable cAMP analog prevents RSV-induced barrier disruption and AJC disassembly

**Fig 2:**
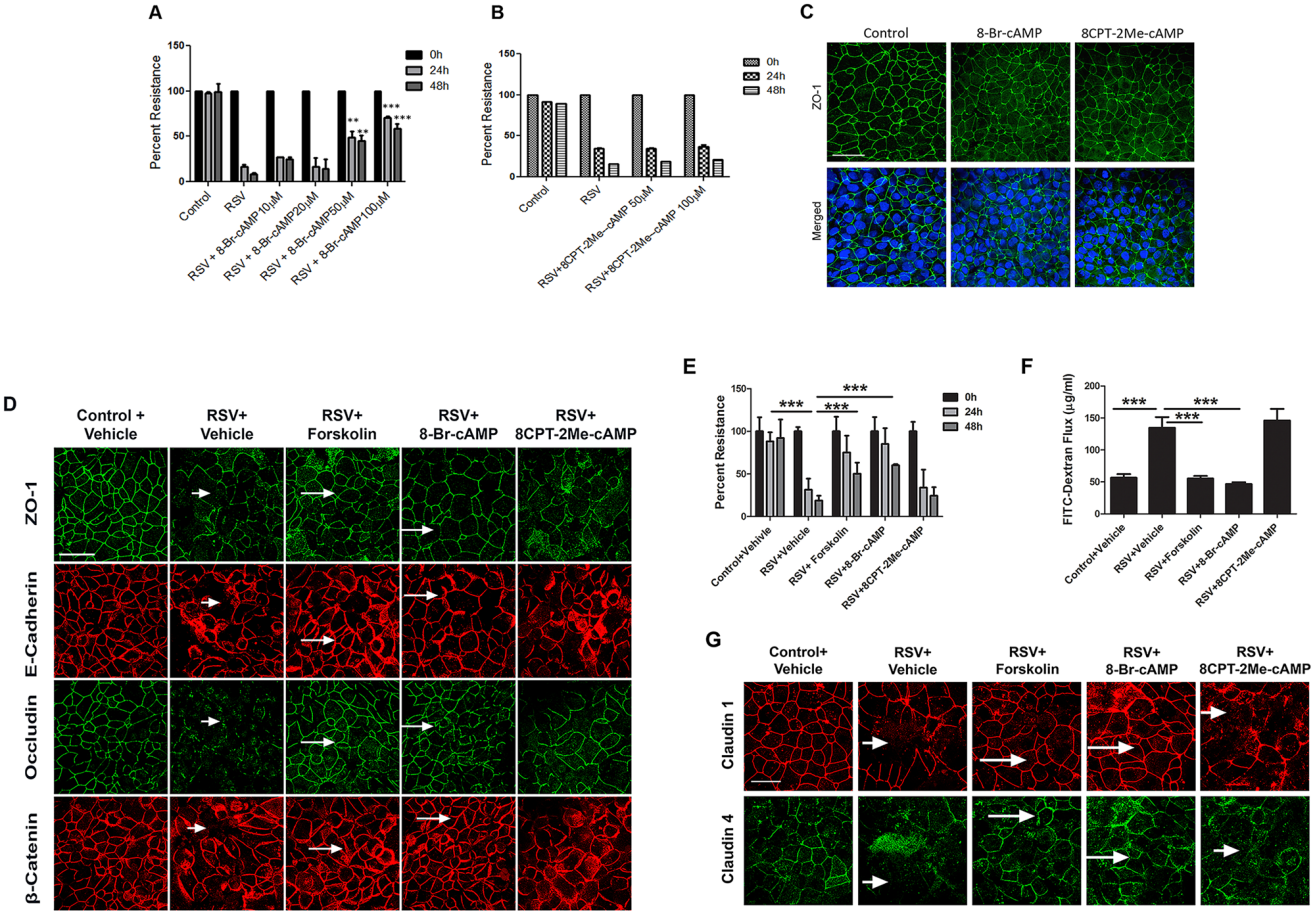
cAMP attenuates RSV-induced epithelial junctional disassembly via Epac-independent mechanisms. (A,B) Confluent epithelial cell monolayers were infected with RSV at an MOI of 0.5 for 48 h in the presence or absence of a stable cAMP analog, 8-Bromo-cAMP (10–100 μM), or an Epac activator, 8CPT-2Me-cAMP (50–100 μM). TEER was measured at indicated time points. (C) Confluent airway epithelial cell monolayers were treated for 48 h with either vehicle, 8-Bromo-cAMP or 8CPT-2Me-cAMP. Cells were fixed and labeled for ZO-1 (green) and nuclei (blue). Scale bar, 40 μm. (D) Airway epithelial cell monolayers were infected with RSV (MOI, 0.5) for 48 h in the presence of the vehicle, forskolin (20 μM), 8-Bromo-cAMP (100 μM), or 8CPT-2Me-cAMP (100 μM). Cells were fixed and immunolabeled for different AJ (E-cadherin, β-catenin) and TJ (ZO-1, occludin) proteins. Note a marked TJ and AJ disassembly in RSV-infected, vehicle treated cells (short arrows) and preservation of normal AJC appearance in virus-infected cells treated with either forskolin or 8-Bromo-cAMP (long arrows). Scale bar, 40 μm. (E, F) Cells were infected with RSV in the presence of either vehicle, forskolin or cAMP analogs, followed by measuring TEER at the indicated times and transepithelial paracellular flux of FITC-dextran at 48 h of viral infection. (G) Immunofluorescence staining for claudin 1 and 4 in epithelial cells infected with RSV. There is marked TJ and AJ disassembly in RSV-infected cells and virus-infected cells treated with 8CPT-2Me-cAMP (short arrows). There is a preservation of normal AJC appearance in virus-infected cells treated with either forskolin or 8-Bromo-cAMP (long arrows). Scale bar, 40 μm. Each image and graph is representative of at least 3 independent experiments. Data is presented as mean ± SEM **, *P<* 0.01 and ***, *P<* 0.001 as compared to RSV-infected vehicle-treated cells.

Since the observed barrier-stabilizing effects of forskolin correlated with the increased production of cAMP, we sought to determine if cAMP analogs could also play protective roles in RSV-infected cell monolayers. Furthermore, we asked if forskolin and cAMP-dependent stabilization of the airways epithelial barrier involve activation of its major effector molecule, Epac. In order to answer these questions, we used 8-Bromo-cAMP (a cell-permeable cAMP analog resistant to degradation by phosphodiesterases) and 8CPT-2Me-cAMP, which is a cell-permeable selective activator of Epac [40]. A dose-response study demonstrated that 8-Bromo-cAMP (50 and 100 μM) consistently attenuated the RSV-induced drop in TEER of bronchial epithelial cell monolayers (Fig 2A). In contrast, the same concentrations of 8CPT-2Me-cAMP, which are known to efficiently activate Epac [40], failed to prevent the RSV-induced reduction in TEER (Fig 2B). Of note, neither of these compounds was toxic to the cells based on the LDH release assay (data not shown). Similar to forskolin, 8-Bromo-cAMP and 8CPT-2Me-cAMP did not affect the integrity of TJ in control bronchial epithelial cell monolayers (Fig 2C). During 48 h of RSV infection, addition of 8-Bromo-cAMP (100 μM) markedly attenuated disruption of TJ (ZO-1 and occludin) and AJ (E-cadherin and β-catenin), thereby mimicking the AJC-protective effects of forskolin (Fig 2D). In contrast, cell exposure to 8CPT-2Me-cAMP failed to attenuate RSV-induced AJC disassembly (Fig 2D). These microscopy data were confirmed by functional studies, where both forskolin and 8-Bromo-cAMP attenuated the RSV-induced drop in TEER and the increased transepithelial dextran flux, whereas 8CPT-2Me-cAMP was ineffective (Fig 2E and 2F). Claudins are integral membrane proteins, known to be components of TJ strands, interacting with other transmembrane TJ proteins, and essential for TJ stability [41, 42]. Consistent with the reported effects on other AJ and TJ proteins, junctional localization of claudin 1 and 4 were disrupted by RSV infection. Both forskolin and 8-Bromo-cAMP, but not 8CPT-2Me-cAMP, attenuated such RSV-induced mislocalization of claudin 1 and claudin 4 in bronchial epithelial cells.

Collectively, these results suggest that forskolin-induced elevation of intracellular cAMP attenuates RSV-induced disruption of the model airway epithelial barrier in an Epac-independent fashion.

### Forskolin induces PKA activation in bronchial epithelial cell monolayers

**Fig 3:**
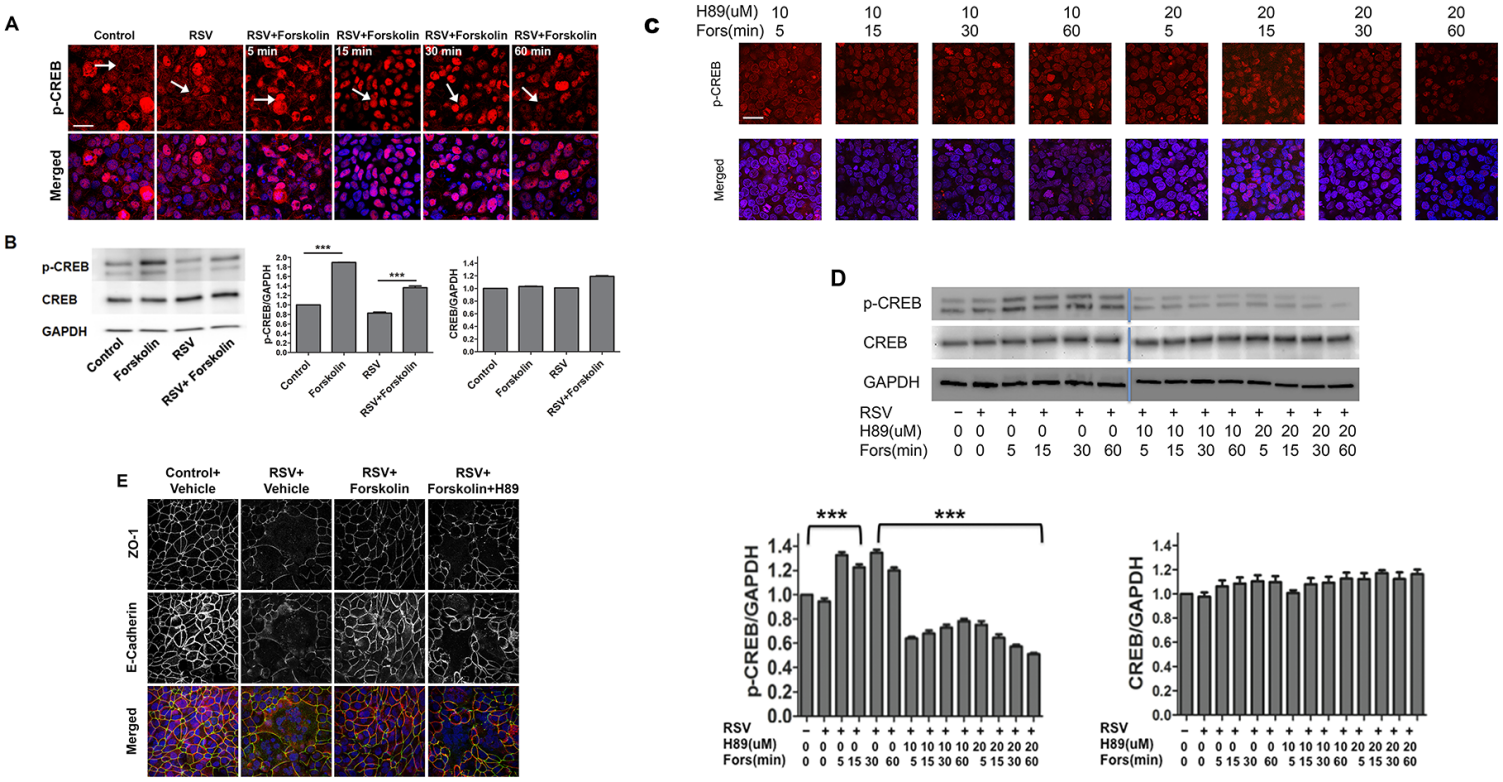
Forskolin-activates PKA signaling in control and RSV-infected airway epithelial cells. Epithelial cell monolayers were infected with RSV (MOI, 0.5), and at 24 h post infection, cells were exposed to forskolin for 5–60 min. (A) Cellular localization of p-CREB (red) was determined by immunolabeling and confocal microscopy. Nuclei were visualized by DAPI labeling (blue). Arrows indicate nuclear localization of p-CREB in forskolin-treated cells. Scale bar, 40 μm. (B) Immunoblot images of p-CREB and total CREB expression, and densitometric quantitative in epithelial cells after 30 min exposure to either vehicle or forskolin, with and without RSV infection for 24 h. (C) Epithelial cells were exposed to H89, a specific PKA inhibitor, for 2 h followed by forskolin treatment for 5–60 min. Cellular localization of p-CREB (red) was determined by immunolabeling and confocal microscopy. Nuclei were visualized by DAPI labeling (blue). Scale bar, 40 μm. (D) Immunoblot images and densitometric quantitative of p-CREB and total CREB expression in epithelial cells after exposure to either vehicle, forskolin, or a combination of forskolin and H89 with and without RSV infection for 24 h. (E) Confluent airway epithelial cell monolayers were infected with RSV (MOI, 0.5) for 48 h, in the presence or absence of forskolin (20 μM), and H89 (10 μM) followed by immunofluorescent staining. The nuclei were counterstained with DAPI (blue). Scale bar, 40 μm. Data is presented as mean ± SEM. Each image and graph is representative of at least 3 independent experiments. Densitometric quantification was performed of 3 independent experiments.

The lack of barrier-protective effect of the pharmacologic Epac activator suggests that forskolin and cAMP attenuate the effects of RSV on epithelial junctions by activating PKA. To test this suggestion, we measured the effects of forskolin on phosphorylation and activation (nuclear translocation) of CREB (cAMP response element-binding) protein, which is a known direct substrate of PKA [43]. Polarized bronchial epithelial cell monolayers were infected with RSV A2 at an MOI of 0.5 for 24 h followed by stimulation with forskolin (20 μM) for 5–60 minutes. Expression and localization of phosphorylated (p), and total, CREB were examined by immunoblotting and immunofluorescence labeling and confocal microscopy. A lower proportion of nuclei exhibited positive labeling for p-CREB in vehicle-treated controls as compared to forskolin-exposed cells, indicating an activation of PKA by this treatment (Fig 3A). Furthermore, forskolin treatment increased the amount of p-CREB but not total CREB in total cell lysates obtained from either control or RSV-infected epithelial cells (Fig 3B). Interestingly, a selective PKA inhibitor, H89, significantly blocked forskolin-induced phosphorylation and nuclear translocation of CREB (Fig 3C and 3D), thereby providing additional evidence of PKA activation in forskolin-treated bronchial epithelial cells. Because H89 attenuated the phosphorylation of CREB, we sought to investigate whether H89 is able to attenuate the protective effect of forskolin on airway barrier. Polarized bronchial epithelial cell monolayers were infected with RSV A2 at an MOI of 0.5 for 48 h in the presence or absence of forskolin (20 μM) and H89 (10 μM). In cells treated with H89, forskolin failed to attenuate RSV-induced AJC disassembly (Fig 3E).

### Elevated cAMP reduces RSV F mRNA in epithelial cells

**Fig 4:**
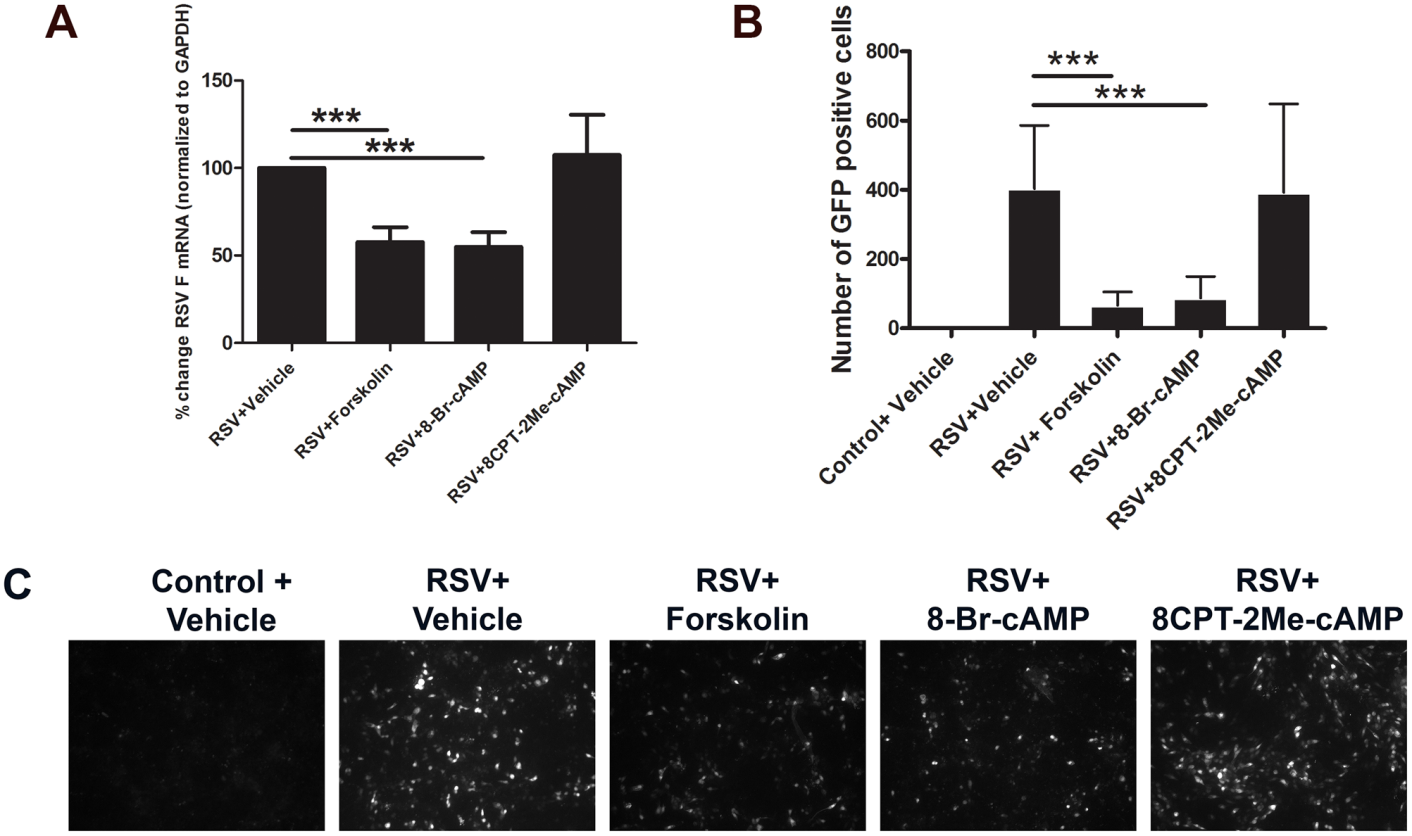
Increase in intracellular cAMP inhibits decreased RSV A2 F mRNA in epithelial monolayers. (A) Polarized epithelial cells were infected with RSV or rgRSV (MOI, 0.5), for 48 h in the presence of the vehicle, forskolin, or cAMP analogs. RSV A2 F mRNA in epithelial cell monolayers was determined by RT-PCR analysis. (B, C) GFP-positive rgRSV-infected cells were visualized and counted by immunofluorescence microscopy. Each image is representative of at least 3 independent experiments. Data is presented as mean ± SEM (n = 3) **, *P<* 0.01 and ***, *P<* 0.001 as compared to RSV-infected vehicle-treated cells.

The infectious cycle of RSV consists of attachment and entry into the host cell, transcription, replication, assembly, and release of new viral particles. During viral entry into epithelial cells, RSV F protein mediates fusion of the virus to the cell membrane, and subsequent cell-to-cell spread by fusion of neighboring cell membranes resulting in the formation of syncytia. Since both forskolin and 8-Bromo-cAMP decreased RSV-induced syncytia formation, we hypothesized that elevated intracellular cAMP, and its downstream signaling effectors, might decrease RSV F mRNA titer. To test this possibility, bronchial epithelial monolayers were infected with RSV (MOI, 0.5) in the presence or absence of forskolin or cAMP analogs, and expression of a viral 87 bp sequence encoding the F protein was quantified by real-time PCR. We found that expression of RSV A2 F mRNA significantly decreased in epithelial cells pretreated with either forskolin or 8-Bromo-cAMP, while 8CPT-2Me-cAMP did not affect RSV A2 F expression (Fig 4A). In a parallel experiment, cells were infected with rgRSV (RSV derived from RSV A2 expressing the green fluorescent protein gene) followed by numeration of GFP-positive cells in the entire monolayer at 22 h post infection. Cells infected with rgRSV and co-treated with forskolin or 8-Bromo-cAMP, but not 8CPT-2Me-cAMP, yielded fewer GFP-positive cells (Fig 4B and 4C). Thus, along with PKA’s preservation of AJC structure and functions, these results suggest that increase in cAMP level inhibits mRNA expression of the F protein and attenuated viral spreading in airway epithelial cell monolayers.

### Forskolin prevents RSV-induced AJC disassembly even when added after viral inoculation

**Fig 5:**
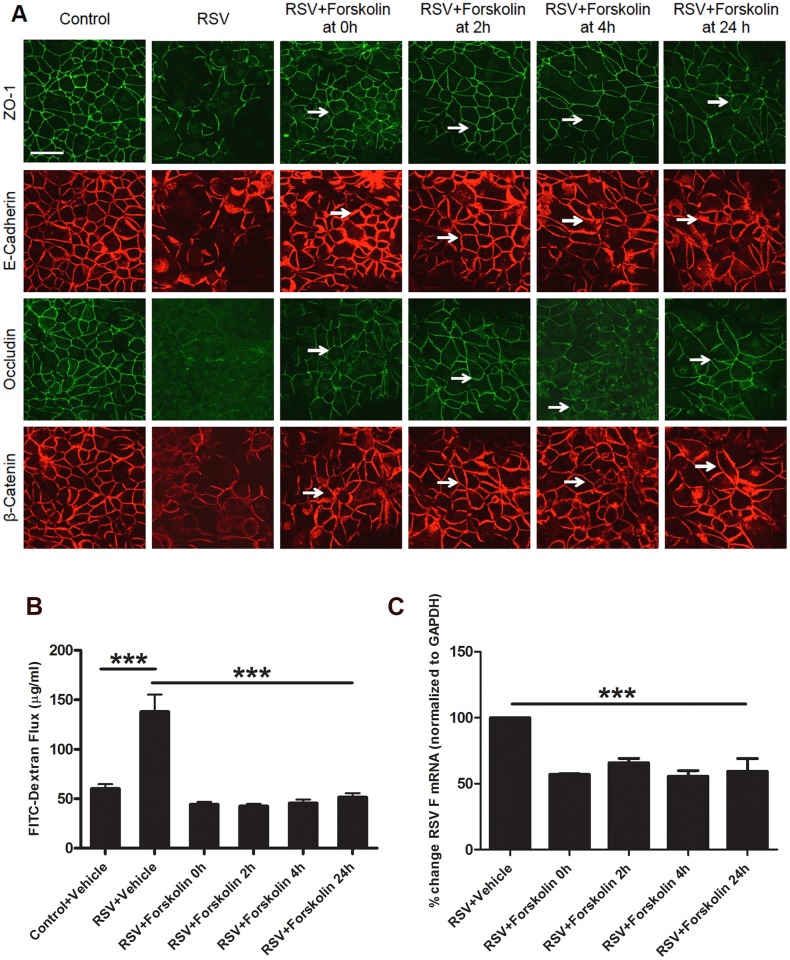
Forskolin exerts protective effects in epithelial monolayers being added after the beginning of RSV infection. Confluent airway epithelial cell monolayers were infected with RSV (MOI, 0.5) for 48 h. Forskolin (20 μM) was added to the cells either at the onset of viral infection (0 h) or at different times after RSV administration. (A) Cells were fixed and immunolabeled for different TJ and AJ proteins. Note that forskolin attenuated RSV-induced TJ and AJ disruption, even when added after the beginning of the infection (arrows). Scale bar, 40 μm. (B) The effect of forskolin on the permeability of control and RSV-infected cell monolayers was determined by measuring transepithelial dextran flux at 48 h of RSV infection. (C) mRNA expression of RSV F protein in epithelial cell lysates was quantified by RT-PCR at 48 h after RSV administration. For all assays, data is presented as mean ± SEM (n = 3). ***, *P*<0.001. Each image is representative of at least 3 independent experiments.

To gain additional insights into the effects of elevated cAMP on the viral infection, we infected cells with RSV (MOI, 0.5), with subsequent addition of forskolin at different times following viral inoculation (0–24 h). Immunofluorescence labeling and confocal microscopy indicate that forskolin inhibited RSV-induced disruption of the AJC structure when it was added after RSV inoculation (Fig 5A). Interestingly, protective effects of forskolin on the organization of epithelial TJ and AJ was evident even when this cAMP-elevating agent was added as late as 24 h after the beginning of viral infection (Fig 5A, arrows). Likewise, this ‘therapeutic’ mode of forskolin addition attenuated the RSV-dependent increase in transepithelial dextran flux (Fig 5B) and inhibited mRNA expression of RSV F protein (Fig 5C).

### cAMP attenuates disruption of the airway epithelial barrier triggered by dsRNA

**Fig 6:**
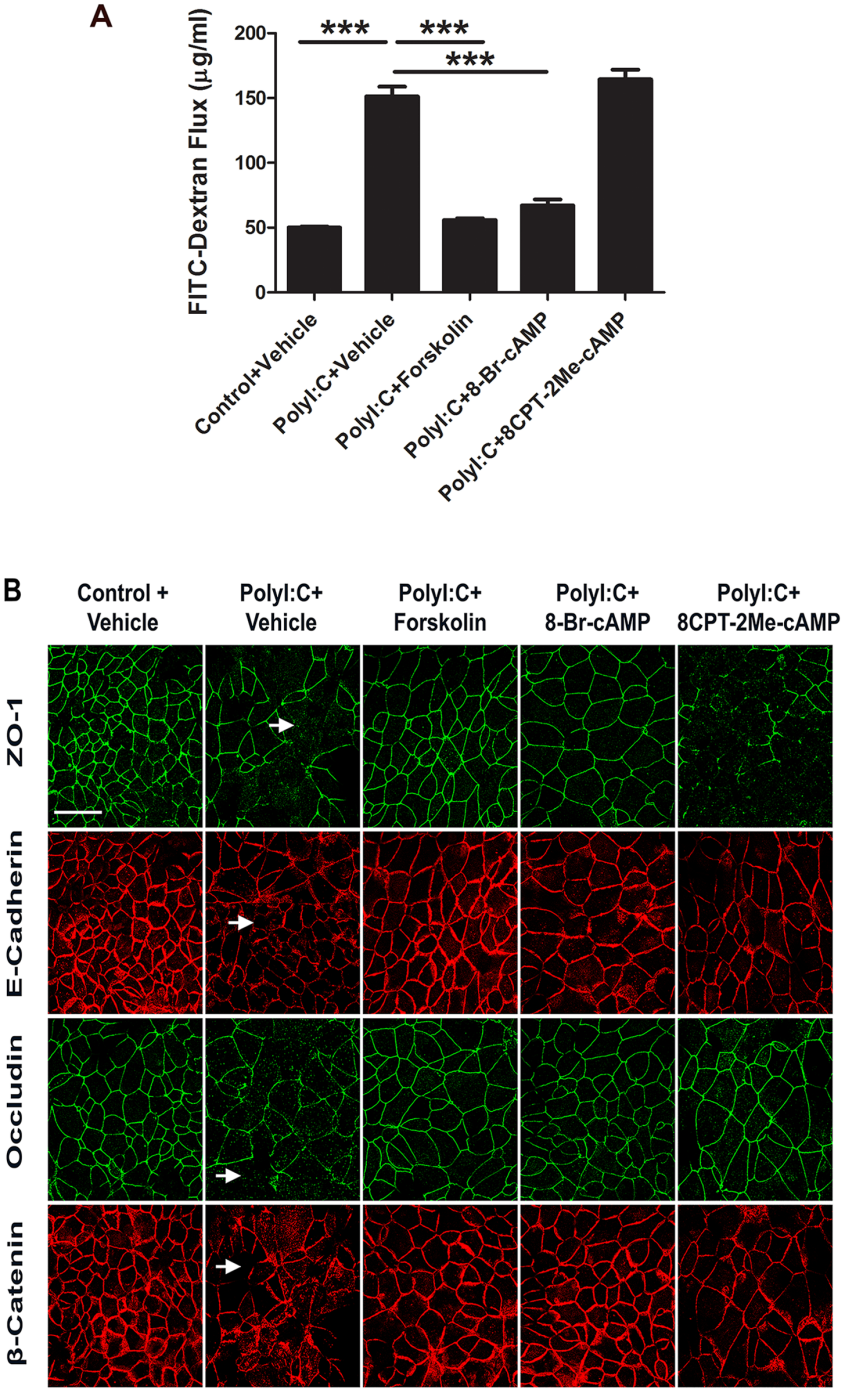
Increase in cAMP level attenuates polyI:C induced disruption of the airway epithelial barrier. Confluent airway epithelial cell monolayers were treated for 24 h with polyI:C (5 μg/ml) in the presence or either vehicle, forskolin (20 μM), or cAMP analogs (100 μM). (A) Barrier permeability was determined by measuring transepithelial dextran flux. Data is presented as mean ± SEM (n = 3); ***, *P<* 0.001. (B) The structure of the epithelial AJC was determined by immunofluorescence labeling and confocal microscopy of different TJ and AJ proteins. Note the disruption in normal TJ and AJ labeling pattern after polyI:C exposure (short arrows), and preservation of normal junction labeling in polyI:C-treated cells in the presence of either forskolin or 8-Bromo-cAMP. Scale bar, 40 μm. Image is representative of at least 3 independent experiments.

The results obtained in this study emphasize two different effects of increased cAMP level in RSV-infected airway epithelial cells. One effect is the attenuation of virus-induced barrier breakdown and AJC disassembly, and the other effect is the inhibition of RSV F mRNA expression and viral propagation within the epithelial monolayers. This creates an important question whether the described barrier-protecting effects of cAMP represent an independent mechanism or is strictly a secondary effect of the inhibited viral propagation. To answer this question, we utilized a synthetic, non-replicating, double-stranded RNA viral mimic (dsRNA Polyinosinic:polycytidylic acid or polyI:C) [44–47]. In agreement with our previous study [34], polyI:C treatment of airway epithelial cell monolayers dramatically increased epithelial permeability (Fig 6A) and caused TJ and AJ disassembly (Fig 6B, short arrows). These effects mimicked barrier-disrupting consequences of RSV infection. The described structural and functional defects of the AJC in polyI:C-treated airway epithelial cells were significantly attenuated by forskolin and 8-Bromo-cAMP treatment, but not 8CPT-2Me-cAMP (Fig 6A and 6B). These results indicate that cAMP can attenuate epithelial barrier dysfunctions caused by viral products by mechanisms independent of inhibition of viral replication.

### Forskolin inhibits RSV-induced remodeling of the cortical F-actin cytoskeleton

**Fig 7:**
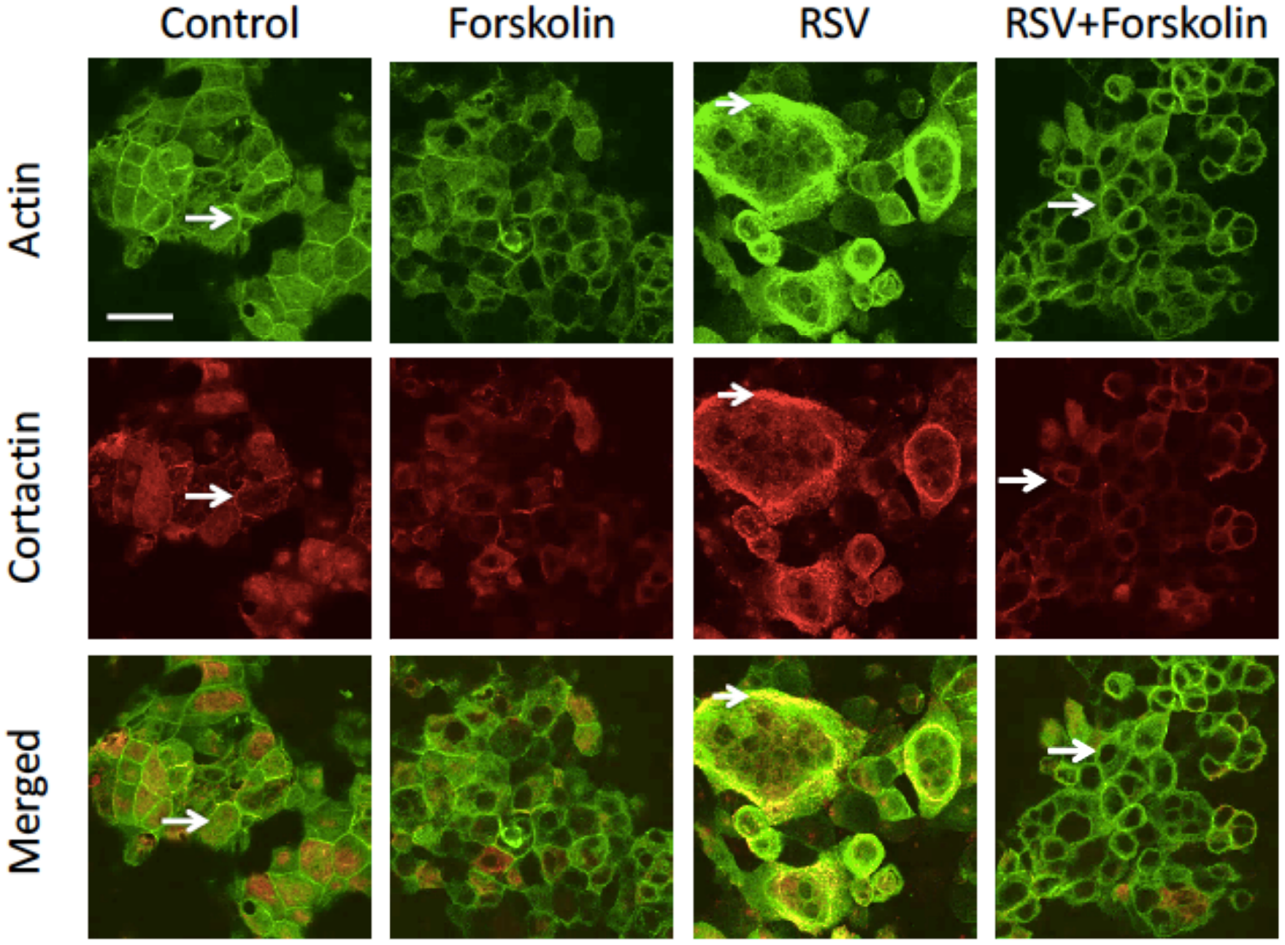
Forskolin prevents RSV-induced remodeling of the perijunctional actin cytoskeleton. Confluent airway epithelial cell monolayers were either left untreated or infected with RSV for 48 h in the presence of either vehicle or forskolin (20 μM). Cells were fixed and labeled for F-actin or cortactin. Note that RSV infection caused the appearance of disorganized apical actin filaments and increased apical cortactin labeling (arrowheads). All these cytoskeletal alterations were attenuated by forskolin treatment (arrows). Scale bar, 40 μm. Image is representative of at least 3 independent experiments.

The integrity and permeability of different epithelial barriers are regulated by the perijunctional actin cytoskeleton [48]. Our previous study demonstrated that RSV infection disrupted the actin cytoskeleton in airway epithelial cell monolayers [19]. Therefore, we next asked whether the observed barrier-protective effects of cAMP are associated with altered remodeling of the junction-associated filamentous (F) actin. Fluorescence labeling was used to visualize F-actin and an essential actin binding protein, cortactin, in control and RSV-infected cell monolayers. Control epithelial cells demonstrated assembly of the prominent circumferential F-actin belt at the level of the AJC (Fig 7, arrows). Cortactin weakly labeled this belt and demonstrated additional apical labeling. RSV infection caused dramatic cytoskeletal rearrangements manifested by the increased accumulation of diffuse actin filaments at the apical region of the cell, especially in the areas of formed syncytia (Fig 7, arrowheads). Cortactin appears to have accumulated at these prominent apical F-actin structures. Interestingly, forskolin, while having little effect on the normal cytoskeleton, completely prevented F-actin remodeling and cortactin translocation in RSV-infected epithelial cells (Fig 7, arrows). These results demonstrate that the observed barrier-protective actions of cAMP could be mediated by the inhibition of cytoskeletal rearrangement in RSV-infected epithelial cell monolayers.

## Discussion

Emerging evidence indicates a critical role for the airway epithelial barrier in regulating responses to environmental stimuli such as allergens and viral infections [49, 50]. While RSV infection causes airway inflammation [9–13] and disrupts the epithelial barrier [19], causal connections between such barrier dysfunctions and propagation of mucosal inflammation have yet to be established.

In the present study, we describe a previously unanticipated pharmacologic approach to stabilize the airway epithelial barrier and limit RSV infection *in vitro*. This approach involves increasing intracellular cAMP level by either applying a pharmacological adenylyl cyclase activator, forskolin, or by using a stable cell-permeable analog of cAMP. The increased cAMP attenuated all detrimental effects of RSV infection on the epithelial barrier, which include increased permeability to ions and large non-charged tracers, as well as TJ and AJ disassembly (Figs 1 & 2). Interestingly, such barrier-protective effects were observed when cAMP was elevated in either ‘prevention’ mode, on the onset of RSV infection, or in the ‘therapeutic’ mode, several hours after initiation of the viral infection (Fig 5). The latter observation is especially important because it raises an attractive possibility of using cAMP-elevating drugs to stabilize the respiratory barrier and limit airway inflammation in RSV-infected patients.

cAMP/PKA signaling was previously implicated in the regulation of epithelial and endothelial barriers, and both barrier-stabilizing and destabilizing roles of this signaling were described [51–54]. Our study also provides insights into the mechanisms by which cAMP protects the epithelial barrier in infected cell monolayers. Specifically, we identified PKA as a key downstream effector of cAMP actions. This conclusion is based on biochemical evidence of PKA activation (phosphorylation and nuclear translocation of Ser133-phosphorylated CREB, Fig 3) in forskolin-treated epithelial cells, as well as on ruling out the involvement of the alternative cAMP effector, Epac (Figs 2 & 6). Additionally, PKA inhibition by H89 blocked forskolin-induced Ser133 phosphorylation of CREB and reversed stabilizing effects of forskolin on apical junctions in RSV-treated bronchial cell monolayers (Fig 3E). PKA phosphorylates a large number of targets including adhesion, cytoskeletal, and signaling proteins, and that different subsets of these proteins are subjected to PKA regulation under different experimental conditions. PKA activation is one of several signaling events triggered by RSV infection. Additionally, RSV is known to induce the activity of the NF-κB signaling pathway [55]. NF-κB is a family of transcription factors that regulate a wide array of genes, particularly those involved with immune responses. The relationship between cAMP and NF-κB is complex, depending on the cell type and treatment. Most papers using epithelial cells report that cAMP inhibits NF-κB activity [56]. However, a few studies have shown either enhanced or constant NF-κB activity with a cAMP inducer [57].

Our data suggest that the actin cytoskeleton is involved in the observed barrier-protective effects of cAMP/PKA signaling in airway epithelial cells. Indeed, both TJ and AJ are known to associate with cortical F-actin bundles [58, 59]. This association is important for the stabilization of TJ and AJ structure in steady-state epithelial cell monolayers. Furthermore, remodeling of the perijunctional actin cytoskeleton drives AJC disassembly and barrier disruption during tissue inflammation [48, 59]. Our present study (Fig 7), along with a previous publication [19], demonstrates that RSV infection triggers reorganization of the perijunctional F-actin that parallels remodeling of apical junctions in airway epithelial cell monolayers. Since RSV does not affect the expression of AJC proteins, we believe it impairs assembly and stability of the AJC by triggering rearrangements of the perijunctional actin cytoskeleton.

The reorganization of actin likely involves altered dynamics (polymerization and depolymerization) of actin filaments, since RSV infection also altered localization (Fig 7), and phosphorylation status [9] of an important regulator of F-actin dynamics, cortactin. Cortactin is a ubiquitously expressed actin-binding protein that mediates F-actin dynamics by promoting filament polymerization [60–62]. Although the roles of cortactin in the regulation of airway epithelial junctions have not been studied, this protein is known to be localized at endothelial junctions in a cAMP-dependent fashion [63]. Immunofluorescence labeling performed in the present study demonstrated diffuse cortical localization of cortactin in control epithelial cells and translocation of cortactin to the lateral plasma membrane in RSV-treated cells. We also noted increased colocalization of cortactin with peripheral F-actin bundles following RSV infection, which may indicate cortactin-dependent remodeling of the actin cytoskeleton that destabilizes the apical junction complex. Of note, the described cytoskeletal remodeling and cortactin relocalization in RSV-infected epithelial cells are likely to be associated with syncytium formation, since these changes were most prominent in the large multinucleated cells (Fig 7).

The exact mechanisms underlying the PKA-dependent regulation of the actin cytoskeleton remain poorly understood, yet several scenarios can be envisioned. One scenario involves direct PKA-dependent phosphorylation of different actin-binding proteins. Examples of such PKA targets are vasodilator-stimulated phosphoprotein that elongates actin filaments [64] and alpha-adducin that caps and bundles actin filaments [65]. Another scenario involves indirect effects of PKA on actin filament dynamics by modulating the activity of small GTPases. Indeed, PKA is known to control the activity of Rac1 and RhoA, both of which are critical regulators of the perijunctional actin cytoskeleton [66, 67]. Further studies are required to determine the exact mechanisms that underlie the protective effects of cAMP/PKA signaling on the actin cytoskeleton and apical junctions in RSV-infected airway epithelial cells.

The pathophysiologic implications of RSV-induced airway epithelial barrier dysfunction are not well understood. Epidemiological studies have repeatedly shown an association between RSV infection, subsequent recurrent wheeze, and chronic airway inflammation. Studies have indicated that E-cadherin levels in asthmatic patients’ sputum correlate with asthma severity [68]. Loss of p120 catenin, an AJ protein, in intestinal epithelial cells was associated with increased mucosal inflammation and intestinal bleeding [69]. Therefore, an important consequence of disrupted integrity of the airway epithelial barrier could be increased tissue inflammation [70]. A dysfunctional epithelial barrier would likely enhance permeability to allergens and particles where they could then encounter dendritic cells and other immune cells, resulting in initiation of immune responses [71]. Inflammation could further exacerbate AJC dysfunction, therefore resulting in a vicious cycle in the airway [71, 72]. Additionally, viral infections have long been known to increase the susceptibility to infection by other pathogens [73, 74]. There is evidence that treatment of influenza infection by an oral neuraminidase prevents mortality from secondary bacterial infections [75]. Therefore, another implication of disrupted epithelial barrier function could be the facilitation of bacterial translocations across epithelial monolayers resulting in secondary infections [38, 76].

Another important finding of this study is that an increase in cAMP level inhibits expression of RSV F protein and viral propagation in the airway epithelial cell monolayers (Figs 4 & 5). RSV is an RNA virus that contains 10 genes encoding for 11 proteins, including the F protein, which promotes syncytia formation and is important in the viral replication cycle. Our study suggests that an elevated intracellular cAMP level decreased both RSV F mRNA and syncytia formation. This is particularly important, as some studies have shown that the degree of viral load plays a critical role in disease severity [77–79], while others report viral load did not correlate with RSV disease severity [80, 81]. In a mouse model of RSV infection, viral load in the respiratory tract was directly correlated with the systemic chemocytokine response, airway inflammation, and respiratory function [77, 82]. Furthermore, DeVincenzo et al. showed in both naturally infected infants and healthy adult volunteers that RSV RNA loads were associated with disease severity [78, 79]. It is reasonable to suggest that inhibition of viral biogenesis contributes to the observed barrier-stabilizing effects of cAMP. However, this is not the only mechanism of cAMP actions, given the fact that this signaling messenger also protects the epithelial AJC against disruption by polyI:C in viral replication-independent fashion (Fig 6). There are few studies on the potential effect of cAMP activators on viral replication. For example, activation of either cAMP/PKA- or Epac/Rap1-dependent signaling has been shown to inhibit HIV-1 replication and cell-to-cell HIV-1 transfer [83, 84]. Pretreatment with cAMP analogs in rat oligodendrocytes inhibited the replication of JHM virus [85]. On the other hand, selective inhibition of Epac significantly reduced susceptibility to Middle East respiratory syndrome coronavirus (MERS-CoV) infections [86]. Furthermore, replication of mammary tumor virus-like particles is stimulated by cAMP, whereas replication of adenovirus can be either inhibited or stimulated by cAMP [62, 87]. Overall, our present study and the described publications highlight cAMP as a potent modulator of viral infections in different tissues and organs.

In summary, this study provides compelling evidence of cAMP/PKA-mediated protection against RSV-induced disruption of the model airway epithelial barrier. Our most striking findings are the multiple targets of cAMP actions in the infected epithelium that include the stabilization of the AJC and the inhibition of RSV propagation. Furthermore, these antiviral and barrier-protective functions of cAMP can be efficiently executed at different stages of the RSV infection, including late stages of RSV propagation within the epithelium. Scientific insights gained from these data will likely guide the design of future therapeutic approaches to treat acute and chronic sequelae of RSV infection. In addition, future studies are planned to further dissect the long-term effects of RSV-induced AJC disruption, persistent lung inflammation, and the role of cAMP signaling pathways. Ultimately, with regards to cAMP/PKA signaling effects, *in vivo* models of RSV infection will be essential to investigate the roles and mechanisms of viral-induced disruption of the pulmonary barrier in the complex physiology of the lungs.

## Conclusions

RSV is a major cause of lower respiratory tract infection and one of the primary reasons for hospitalization worldwide. There is no efficient antiviral therapy or vaccine to manage the disease. The cAMP signaling pathway is involved in the regulation of many essential cellular processes in differentiated epithelial layers. Here we sought to identify the effects of RSV on the integrity of the airway epithelial barrier and the modulation of these effects by cAMP signaling. We found that activation of cAMP prevents RSV-induced airway epithelial barrier dysfunction. Furthermore, cAMP activation decreased RSV viral titer even when treatment was delivered after RSV infection. These findings provide novel insights into understanding epithelial cell responses to a clinically significant virus with poorly defined pathogenesis and limited treatment options.

## Abbreviations

AJ: Adherens junction
AJC: Apical junctional complex
CREB: cAMP response element-binding protein
dsRNA: Double-stranded RNA
E-Cadherin: Epithelial Cadherin
FITC: fluorescein isothiocyanate
pAb: Polyclonal antibody
pCREB: phosphorylated CREB
polyI:C: Polyinosinic:polycytidylic acid
RSV: Respiratory syncytial virus
TEER: Transepithelial electrical resistance
TJ: Tight junction
ZO-1: Zonula occludens protein 1
